# Microbial Communities in Flexible Biomethanation of Hydrogen Are Functionally Resilient Upon Starvation

**DOI:** 10.3389/fmicb.2021.619632

**Published:** 2021-02-11

**Authors:** Washington Logroño, Denny Popp, Marcell Nikolausz, Paul Kluge, Hauke Harms, Sabine Kleinsteuber

**Affiliations:** Department of Environmental Microbiology, Helmholtz Centre for Environmental Research – UFZ, Leipzig, Germany

**Keywords:** power-to-gas, biomethane, biogas upgrading, hydrogenotrophic methanogenesis, wastewater treatment, anaerobic digester, intermittent feeding, *Methanobacterium*

## Abstract

*Ex situ* biomethanation allows the conversion of hydrogen produced from surplus electricity to methane. The flexibility of the process was recently demonstrated, yet it is unknown how intermittent hydrogen feeding impacts the functionality of the microbial communities. We investigated the effect of starvation events on the hydrogen consumption and methane production rates (MPRs) of two different methanogenic communities that were fed with hydrogen and carbon dioxide. Both communities showed functional resilience in terms of hydrogen consumption and MPRs upon starvation periods of up to 14 days. The origin of the inoculum, community structure and dominant methanogens were decisive for high gas conversion rates. Thus, pre-screening a well performing inoculum is essential to ensure the efficiency of biomethanation systems operating under flexible gas feeding regimes. Our results suggest that the type of the predominant hydrogenotrophic methanogen (here: *Methanobacterium*) is important for an efficient process. We also show that flexible biomethanation of hydrogen and carbon dioxide with complex microbiota is possible while avoiding the accumulation of acetate, which is relevant for practical implementation. In our study, the inoculum from an upflow anaerobic sludge blanket reactor treating wastewater from paper industry performed better compared to the inoculum from a plug flow reactor treating cow manure and corn silage. Therefore, the implementation of the power-to-gas concept in wastewater treatment plants of the paper industry, where biocatalytic biomass is readily available, may be a viable option to reduce the carbon footprint of the paper industry.

## Introduction

The growing share of variable renewable energy, mainly photovoltaics and wind power, generates temporary surplus electricity that leads to an energy storage problem. To address this issue, energy storage and flexible energy conversion are required (Strübing et al., 2019). The power-to-gas (P2G) approach is an elegant way to store surplus electricity in the form of a chemical energy carrier that can be consumed independently of electricity production, e.g., hydrogen or methane (Schiebahn et al., 2015). Hydrogen has various applications as fuel and chemical feedstock or can be stored in the natural gas grid though only up to certain limits (Bailera et al., 2017). Methane has also various applications as fuel or chemical building block, but compared to hydrogen it is more compatible with the existing infrastructure. It is easier to store and to transport due to its higher energy density and can be readily injected into the natural gas grid (Blanco et al., 2018).

The production of methane from surplus electricity is carried out in a two-step process: first hydrogen is produced by water electrolysis (Eq. 1), which is then used in the second step to reduce carbon dioxide to methane (Eq. 2) (Schaaf et al., 2014).

(1) $$4\,{\text{H}}_{2}\,\text{O}\to 2\,O_{2}+4\,{\text{H}}_{2}$$

(2) $$4\,{\text{H}}_{2}+{\text{CO}}_{2}\to {\text{CH}}_{4}+2\,{\text{H}}_{2}\,\text{O}$$

Methanation can be performed by a catalyst-based chemical reaction, known as the Sabatier reaction, or in a microbial process employing the CO_2_-reductive pathway of hydrogenotrophic methanogenesis. The latter seems advantageous over the Sabatier reaction in terms of catalyst regeneration and milder process conditions (Angelidaki et al., 2018). *Ex situ* biomethanation is a microbial process that uses point sources of CO_2_ or the CO_2_ fraction of biogas to produce high quality biomethane. It can be carried out by methanogenic pure cultures (Rittmann et al., 2015) or mixed cultures (Angelidaki et al., 2018). In a recent study comparing different reactor systems, the most efficient reactor produced methane of 98% purity (Kougias et al., 2017). During *ex situ* biomethanation with mixed cultures, volatile fatty acids (VFAs) such as acetate and propionate are produced (Burkhardt and Busch, 2013; Alitalo et al., 2015; Burkhardt et al., 2015, 2019; Rachbauer et al., 2016; Kougias et al., 2017; Savvas et al., 2017; Strübing et al., 2017, 2018; Yun et al., 2017; Ullrich et al., 2018). Acetate can be produced from hydrogen and CO_2_ via the Wood–Ljungdahl pathway of acetogenic bacteria (Eq. 3), which competes with hydrogenotrophic methanogenesis for the electron donor and carbon source. However, if acetotrophic methanogens are present in the mixed culture, acetate is eventually converted to methane (Eq. 4) (Angelidaki et al., 2018). Alternatively, acetate can be converted to hydrogen and CO_2_ by syntrophic acetate-oxidizing bacteria (SAOB), provided the hydrogen partial pressure is kept sufficiently low due to immediate consumption (Eq. 5). Hence, this reaction relies on the syntrophic cooperation of SAOB with hydrogenotrophic methanogens (Eq. 2) (Hattori, 2008). Acetate is also assimilated by bacteria and archaea to build microbial biomass.

(3) $$4\,{\text{H}}_{2}+2\,{\text{CO}}_{2}\to {\text{CH}}_{3}\,\mathrm{COOH}+2\,H_{2}\,\text{O}$$

(4) $${\text{CH}}_{3}\,\text{COOH}\to {\text{CH}}_{4}+{\text{CO}}_{2}$$

(5) $${\text{CH}}_{3}\,{\text{COO}}^{-}+4\,{\text{H}}_{2}\,\text{O}\to 2\,H\,C\,O_{3}^{-}+{\text{H}}^{+}+4\,H_{2}$$

Surplus electricity is fluctuating due to seasonal and diurnal changes; therefore, biomethanation requires a flexible process enduring idle periods without performance loss. Recently, the flexibility of the process was systematically investigated with standby periods of 1–8 days and at temperatures of 25 and 55°C (Strübing et al., 2018, 2019). However, the effect of starvation periods on the microbial communities in *ex situ* biomethanation is yet to be investigated. Here, we studied the impact of fluctuations in gas supply causing intermittent starvation of the hydrogenotrophic community in an *ex situ* biomethanation system. Process parameters, community profiles and changes in microbial diversity upon different starvation periods were analyzed in two methanogenic consortia from different origin. Further, we assessed the effect of the inoculum source on the performance of the biomethanation process and propose a concept to implement the P2G approach for biogas upgrading in wastewater treatment plants.

## Materials and Methods

### Experimental Setup

Mesophilic anaerobic granular sludge from an industrial-scale upflow anaerobic sludge blanket (UASB) reactor treating wastewater from paper industry (designated as WW) was sampled and transported to the laboratory under anoxic conditions. The sludge granules were crushed upon inoculum preparation as described in Supplementary Text 1. The second mesophilic inoculum sludge was digestate from a pilot-scale plug-flow reactor digesting cow manure and corn silage (designated as PF). Both inocula were degassed for 5 days at 37°C. Reactor experiments were conducted in four biological replicates under strict anaerobic conditions in serum bottles (219.5 mL) filled with 50 mL of master inoculum mixture (Supplementary Text 1). The master inoculum mixtures were prepared with sludge sieved through 400 μm mesh size in mineral medium containing 0.2 g L^–1^ yeast extract. Medium supplemented with such low concentrations of yeast extract is regarded as mineral medium (Stams et al., 1993). The initial pH was set to 9.0 with a sterile anoxic stock solution of 2 M KOH. The bottles were incubated at 37°C. Liquid samples of 5 mL were withdrawn weekly and an equal volume of fresh medium was added. In the beginning the reactors were operated in fed-batch mode (feeding every 24 h, except weekends) with a gas mixture of H_2_/CO_2_ (4:1) and a total pressure of ∼2.2 bar. In each fed-batch cycle, the gas produced was withdrawn before feeding the gas mixture. Detailed information on flushing and pressurization procedures is given elsewhere (Logroño et al., 2020). The background biogas production from the inoculum was determined by setting up three biological controls with N_2_/CO_2_ (4:1) in the headspace.

**FIGURE 1:**
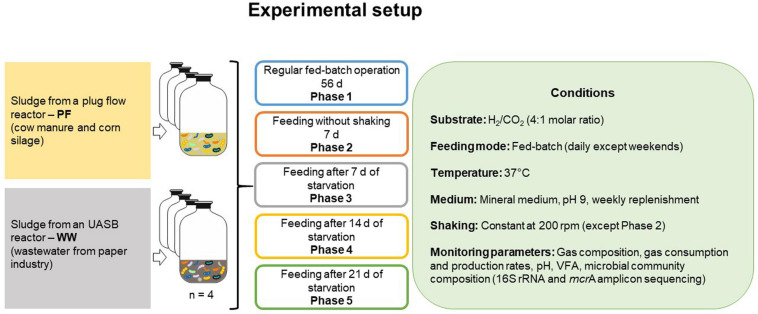
Schematic of the experimental setup. Each inoculum was set up in four biological replicates.

The starvation experiment comprised five phases as illustrated in Figure 1: after a regular fed-batch phase with feeding every 24 h over 56 days (phase 1), one fed-batch cycle without shaking lasted 7 days (phase 2), followed by single fed-batch cycles (24 h each) after starvation periods of 7 days (phase 3), 14 days (phase 4), and 21 days (phase 5). The bottles were shaken at 200 rpm except in phase 2 to determine the effect of gas mass transfer limitation. Gas consumption and production rates were determined at three sampling times during phase 1 (days 7, 21, and 53) and once during each of phases 2–5. Gas amounts are presented in mmol and were calculated as previously described (Logroño et al., 2020). The mean values of gas consumption and production rates in phase 1 were used as a reference for comparison with the other phases. Liquid samples were analyzed at the end of each phase.

### Analytical Methods

The pressure was measured with a high resolution manometer (LEO 5, Keller, Switzerland) as described previously (Logroño et al., 2020). The gas composition was analyzed after every batch cycle in 1 mL gas samples via gas chromatography (GC). The pH value of the broth was recorded in 200 μL samples with a mini-pH meter (ISFET pH meter S2K922, ISFETCOM Co., Ltd., Hidaka, Japan). VFA concentrations in the liquid phase were analyzed by high performance liquid chromatography (HPLC). In brief, 1.5 mL samples were withdrawn, centrifuged at 20,817 × *g* and 4°C for 10 min, and subsequently filtered through cellulose acetate membrane filters with a pore size of 0.22 μm (13 mm; LABSOLUTE, Th. Geyer GmbH, Hamburg, Germany) and stored at −20°C if not measured immediately. Detailed information about the GC and HPLC setup was given in our previous study (Logroño et al., 2020).

### Microbial Community Analysis

Liquid samples (1.5 mL) were taken at the start of the experiment and at the end of each experimental phase. The samples were centrifuged at 4°C and 20,817 × *g* for 10 min. Genomic DNA from the pellet was extracted with the NucleoSpin Soil kit (MACHEREY-NAGEL GmbH & Co., KG, Germany) using buffer SL2 and enhancer solution as indicated in the manufacturer’s protocol. Extracted DNA was stored at −20°C until use. The microbial community composition was analyzed by amplicon sequencing of *mcrA* genes for methanogens and 16S rRNA genes for bacteria.

The V3–V4 region of the 16S rRNA genes was amplified using the primers 341f and 785r (Klindworth et al., 2013). For methanogens, the mlas and mcrA-rev primers were used (Steinberg and Regan, 2008). Amplicon sequencing was performed on the Illumina MiSeq platform using the MiSeq Reagent Kit v3 with 2 × 300 cycles. Bioinformatic analysis was done as described previously (Logroño et al., 2020). In brief, primer sequences were clipped from demultiplexed and adapter-free reads using Cutadapt v1.18 (Martin, 2013), and further sequence analysis was performed with the QIIME2 v2019.1 pipeline (Bolyen et al., 2019) using the dada2 plugin (Callahan et al., 2016). The resulting amplicon sequencing variants (ASVs) for 16S rRNA genes were classified against the MiDAS database v2.1 (McIlroy et al., 2017) trimmed to the region covered by the 341f and 785r primers. Archaeal 16S rRNA reads were removed from the dataset and bacterial read counts were normalized to 100%. For *mcrA* ASVs, a taxonomy database compiled by using *mcrA* sequences from the RDP FunGene database (Fish et al., 2013) was used.

### Microbial Community Ecological Indices

Amplicon sequencing variant data was used to calculate indices quantifying α-diversity as described by Lucas et al. (2017). Briefly, the Shannon index (H) describes the uncertainty to predict the identity of an unknown individual randomly chosen from a community (Eq. 6), richness (R) reflects the number of present types regardless of their particular relative abundances (Eq. 7), diversity of order one (^1^D) quantifies the community diversity by weighting all present types according to their particular abundances (Eq. 8), diversity of order two (^2^D) quantifies the community diversity by weighting the most common types significantly more than the rare types (Eq. 9), evenness of order one (^1^E) reflects the abundance heterogeneity of the types in a community (Eq. 10).

(6) $$H=-\sum_{i=1}^{R}p_{i}\,\ln p_{i}$$

(7) D0=∑i=1Rpi0=R

(8) D1=exp⁡(H)

(9) D2=1⁢/⁢(∑i=1Rpi2)

(10) E1=D1⁢/⁢R=exp⁡(H)⁢/⁢R

To quantify the β-diversity, the microbial community composition data was subjected to principal coordinate analysis (PCoA) based on Bray–Curtis distances using the phyloseq package (Bolyen et al., 2019) version 1.30.0. PCoA was plotted using the ggplot2 package (Wickham, 2016) version 3.2.1.

### Statistical Analysis

Differences between phases were studied by analysis of variance (ANOVA). Tukey’s *post-hoc* test was used for multiple comparisons and significant differences were denoted ^∗^ when *p* < 0.05 [*p* > 0.05 (ns: not significant), *p* < 0.05 (^∗^), *p* < 0.01 (^∗∗^), *p* < 0.001 (^∗∗∗^), and *p* < 0.0001 (^****^)]. Analysis of similarities (ANOSIM) was used to test if the intra-community variation could be explained by the experimental phases and calculated in R (R Core Team, 2019) version 3.6.1 using the Vegan package (Oksanen et al., 2019). Ecological indices were compared between phases with ANOVA and Tukey’s *post-hoc* test as aforementioned. Graphpad (Graphpad Software, Inc., San Diego, CA, United States) or Microsoft Excel were used to compute the data.

## Results and Discussion

### Effects of Starvation on the Process Performance

**FIGURE 2:**
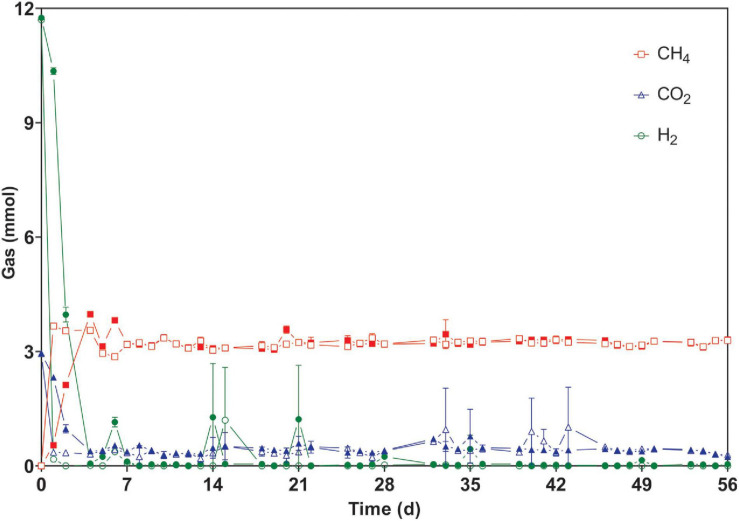
Time course of consumed and produced gases during regular fed-batch operation (phase 1). The bottles were fed every 24 h (except weekends) and measurements were carried out at the end of each batch cycle. Open symbols: reactors WW (inoculated with anaerobic granular sludge from an industrial-scale UASB reactor treating wastewater from paper industry); filled symbols: reactors PF (inoculated with digestate from a pilot-scale plug flow reactor digesting cow manure and corn silage). The mean and standard deviation of *n* = 4 are depicted. Invisible error bars are smaller than the symbol.

After the first feeding, the production of CH_4_ from H_2_/CO_2_ in reactors WW was three times faster than that in PF, as the gaseous substrate was depleted after 24 and 72 h, respectively. Thereafter, all cultures converted the gaseous substrate within ∼24 h in a stable manner for 56 days during phase 1 (Figure 2 and Supplementary Figure 1). Previous studies have also reported complete gas conversion within 24 h (Kern et al., 2016; Logroño et al., 2020).

**FIGURE 3:**
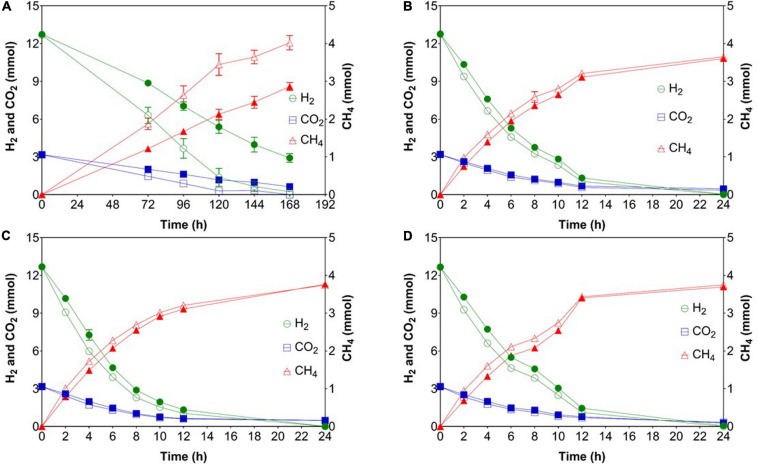
Gas consumption (H_2_ and CO_2_) and production (CH_4_) rates after starvation events. **(A)** Phase 2 (feeding without shaking), **(B)** phase 3 (after 7 days of starvation), **(C)** phase 4 (after 14 days of starvation), **(D)** phase 5 (after 21 days of starvation). Open symbols: reactors WW (inoculated with anaerobic granular sludge from an industrial-scale UASB reactor treating wastewater from paper industry); filled symbols: reactors PF (inoculated with digestate from a pilot-scale plug flow reactor digesting cow manure and corn silage). The mean and standard deviation of *n* = 4 are depicted.

In phase 1, the mean gas consumption (H_2_ and CO_2_) and production (CH_4_) rates of WW were significantly higher than those of PF (Table 1) although the methane content was comparable for both inocula. When shaking was omitted in phase 2, all cultures suffered from gas mass transfer limitations as reflected by extremely low gas consumption and production rates (Table 1). In case of PF, the H_2_ consumption rate in phase 3 and 5 were 2 and 11% lower than in phase 1. Similarly for WW, the H_2_ conversion rate in phase 3 and 5 were 5 and 11% lower than in phase 1. Interestingly, the highest H_2_ consumption rates were observed in phase 4 for both WW and PF (after 14 days of starvation) (Table 1). The trends of consumption and production rates in phases 2–5 are shown in Figure 3.

**TABLE 1:** Summary of process performance during different experimental phases.

				**H_2_**	**CO_2_**	**CH_4_**	**H_2_ consumption**
**Inoculum**	**Phase**	**pH**	**CH_4_ (%)**	**(mmol L^–1^ h^–1^)**	**(mmol L^–1^ h^–1^)**	**(mmol L^–1^ h^–1^)**	**efficiency (%)**
WW	Start	9.02 ± 0.00	–	–	–	–	–
	1 (56 days)	8.29 ± 0.03	97.56 ± 0.21^a^	25.02 ± 0.28^b^ (****)	5.81 ± 0.08^b^ (*)	6.73 ± 0.09^b^ (*)	98.3 ± 10.3
	2 (7 days)	8.36 ± 0.04	91.66 ± 5.10	1.56 ± 0.03 (****)	0.38 ± 0.00 (****)	0.50 ± 0.01 (****)	98.3 ± 1.8
	3 (7 days)	8.18 ± 0.03	88.53 ± 0.58	23.83 ± 0.48 (****)	5.21 ± 0.14 (****)	6.34 ± 0.29 (**)	100.0 ± 0.0
	4 (14 days)	8.18 ± 0.03	88.80 ± 0.96	25.91 ± 0.34 (**)	5.54 ± 0.03 (**)	6.65 ± 0.15	100.0 ± 0.0
	5 (21 days)	8.08 ± 0.03 (*)	91.93 ± 0.71	22.26 ± 0.30 (****)	5.06 ± 0.10 (****)	5.83 ± 0.11 (****)	100.0 ± 0.0
PF	Start	9.00 ± 0.00	–	–	–	–	–
	1 (56 days)	8.39 ± 0.04	96.57 ± 0.23^a^	23.46 ± 0.20	5.61 ± 0.06	6.42 ± 0.05	97.6 ± 11.6
	2 (7 days)	8.06 ± 0.22 (****)	44.81 ± 3.57	1.20 ± 0.06 (****)	0.31 ± 0.01 (****)	0.34 ± 0.02 (****)	77.6 ± 2.7
	3 (7 days)	8.12 ± 0.10 (**)	87.79 ± 1.54	23.02 ± 0.23	4.96 ± 0.06 (****)	5.21 ± 0.14 (***)	99.7 ± 0.5
	4 (14 days)	8.00 ± 0.06 (****)	88.66 ± 1.28	25.09 ± 0.06 (****)	5.41 ± 0.10 (*)	6.39 ± 0.07	100.0 ± 0.0
	5 (21 days)	8.17 ± 0.04 (*)	91.69 ± 0.91	20.94 ± 0.24 (****)	4.78 ± 0.10 (****)	5.35 ± 0.10 (****)	100.0 ± 0.1

ND: not detected. The mean and standard deviation of *n* = 4 are given. Significant differences between phase 1 and other phases are indicated with * (*p* < 0.05), ** (*p* < 0.01), *** (*p* < 0.001) or **** (*p* < 0.0001) considering *n* = 4. ^*a*^The highest methane concentration in phase 1 is presented. The values of the other parameters were determined at the end of each phase. ^*b*^Comparison between the rates of WW and PF in phase 1 (regular fed-batch).

Comparing the methane production rates (MPRs) in phase 1 (regular fed-batch) to those in phase 3 (7 days of starvation), phase 4 (14 days of starvation), and phase 5 (21 days of starvation) showed that both communities responded in a similar way (Table 1). The rates in phase 1 were significantly higher than those in phase 3 and 5. No differences in MPR between phase 1 and 4 (14 days of starvation) were observed, suggesting functional resilience of the microbiota. However, the MPR in phase 5 (21 days of starvation) was significantly lower than in phase 1, which may reflect the limits of resilience.

The pH values decreased from 9 to ∼8.3 in all cultures (Table 1). Previous studies where the pH was not controlled and the initial experimental pH was lower than in our study have also shown that the system stabilized to similar pH values (8–8.5) after H_2_/CO_2_ feeding (Bassani et al., 2017; Kougias et al., 2017; Logroño et al., 2020). When comparing the phase of regular feeding (phase 1) to the other phases, the two communities revealed a different behavior. In case of PF, the pH values in phases 2–5 were significantly lower than in phase 1 (Table 1). With WW, the pH did not drop significantly over phases 1–4 but the pH in phase 5 was significantly lower than in phase 1 (Table 1). Recently it was reported that pH variations change the energy yields of redox reactions under anoxic conditions (here: methanogenesis) and thereby can influence the structure and function of microbial communities (Jin and Kirk, 2018).

Contrary to previous findings where acetate and propionate were produced (Savvas et al., 2017; Strübing et al., 2018), only traces of VFAs (below 10 mg L^–1^) were detected in our experiments despite using complex microbiota. Several explanations for this observation are conceivable: Either homoacetogens could not compete with hydrogenotrophic methanogens for H_2_ and thus only low amounts of acetate were produced, or acetate produced by homoacetogens during sufficiently high H_2_ levels was readily converted to CH_4_ by acetotrophic methanogens, or by SAOB when the H_2_ partial pressure was sufficiently low. The H_2_ consumption efficiency was ∼100% in all phases except for PF in phase 2, when gas mass transfer limitation was reflected by the lowest H_2_ consumption rates (Table 1 and Figure 3). Such high H_2_ consumption efficiency is consistent with the findings reported earlier (Strübing et al., 2018).

### Effects of Starvation on the Microbial Community Diversity

**FIGURE 4:**
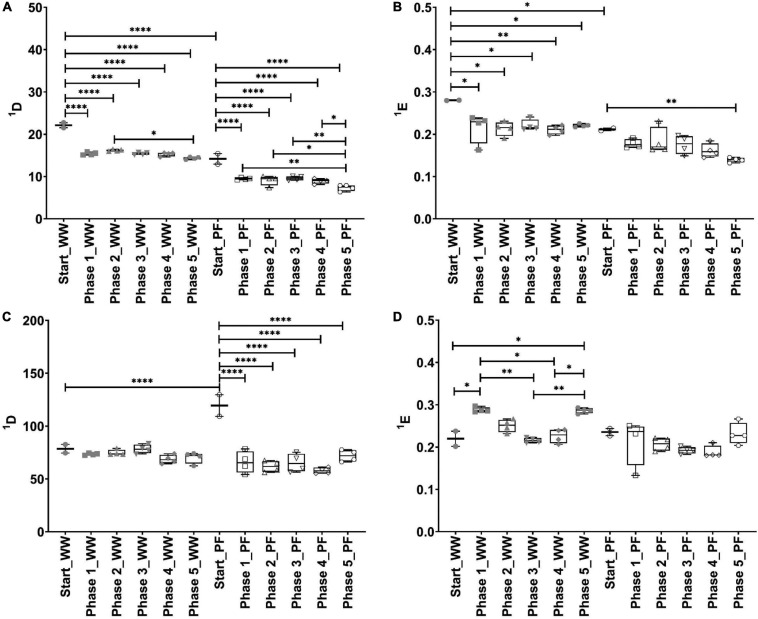
Box plots of diversity **(A,C)** and evenness **(B,D)** for *q* = 1 in different phases. Methanogenic communities **(A,B)** and bacterial communities **(C,D)**. Significant differences between groups are indicated with * when *p* < 0.05, ** when *p* < 0.01, and **** when *p* < 0.0001. Note that for start *n* = 2 whereas for phases 1 to 5 *n* = 4. WW: reactors inoculated with anaerobic granular sludge from an industrial-scale UASB reactor treating wastewater from paper industry; PF: reactors inoculated with digestate from a pilot-scale plug-flow reactor digesting cow manure and corn silage.

The rarefaction curves from amplicon sequencing analysis of *mcr*A and 16S rRNA genes indicated that sufficient sequencing depth was reached in all samples (Supplementary Figure 2). To evaluate the α-diversity in the single phases of each community, we calculated the diversity and evenness of order one (Figure 4). ANOVA revealed significant differences in both indices for both bacteria and methanogens.

The methanogenic community from the start phase of the WW inoculum was significantly more diverse than that from the PF inoculum (*p* < 0.0001) (Figure 4A). Feeding stoichiometric H_2_:CO_2_ ratio required for hydrogenotrophic methanogenesis led to a significant decrease in diversity, which is evident when comparing the start phase to the other phases in both inocula. However, it is to note that even after starvation events no significant diversity changes in the methanogenic and bacterial communities were found when comparing phase 1 to the other phases. In the start phase, evenness of the WW methanogenic community was significantly higher (*p* = 0.0345) than that of the PF methanogenic community (Figure 4B). Evenness significantly decreased for WW when comparing the start phase to the other phases but significant differences for PF were only observed between start and phase 5 (*p* = 0.0026). Additional α-diversity indices such as H, R, and ^2^D of the methanogenic community are presented in Supplementary Figures 3A–C.

Comparing diversity of order one (^1^D) from the start phase of WW and PF indicated that the bacterial community of PF was significantly more diverse than that of the WW inoculum (*p* < 0.0001) (Figure 4C). These differences can be explained by the low abundant taxa rather than the most abundant ones since diversity of order two (^2^D) was not different between the start phases of the WW and PF inocula (Supplementary Figure 3F). Furthermore, ^1^D remained unchanged for WW but it significantly decreased for PF when comparing the start phase to the other phases. Figure 4D shows that evenness of the bacterial communities was comparable for both inocula in the start phase. Evenness of the WW bacterial community was significantly higher than that of the PF bacterial community in phase 1 and 5 but not in the other phases. Other α-diversity indices (H, R, and ^2^D) of the bacterial community are presented in Supplementary Figures 3D–F.

The functional resilience, i.e., the ability of microbial communities to return to a stable process state after a disturbance, was evaluated in terms of MPR. MPRs in phase 3 and 5 were significantly lower than in phase 1 for both inocula, indicating that sudden and extended disturbances result in a decreased performance, which is explained by lower resilience of the microbial communities. Considering that the MPR of WW was higher than that of PF (Table 1) even after starvation events, it can be hypothesized that this is linked to the type of hydrogenotrophic methanogens present in the community. By comparing diversity ^2^D (which gives more weight to the most abundant taxa) between inocula for each independent phase we found that the diversity of the WW methanogenic community was significantly higher than that of the PF methanogenic community (Supplementary Figure 3C; *p* < 0.0001 for all comparisons). Therefore, it appears that the diversity of methanogens may play a role in coping with disturbances since the more diverse community (WW) was more functionally resilient than PF. A recent study by Figeac et al. (2020) evaluated the effect of temperature and origin of the inoculum on the performance and stability of *ex situ* biomethanation with mixed cultures. Although MPR of the thermophilic process was higher, the instability of the process increased along with temperature due to the unique dominance of *Methanothermobacter*.

### Effects of Starvation on the Microbial Community Structure

**FIGURE 5:**
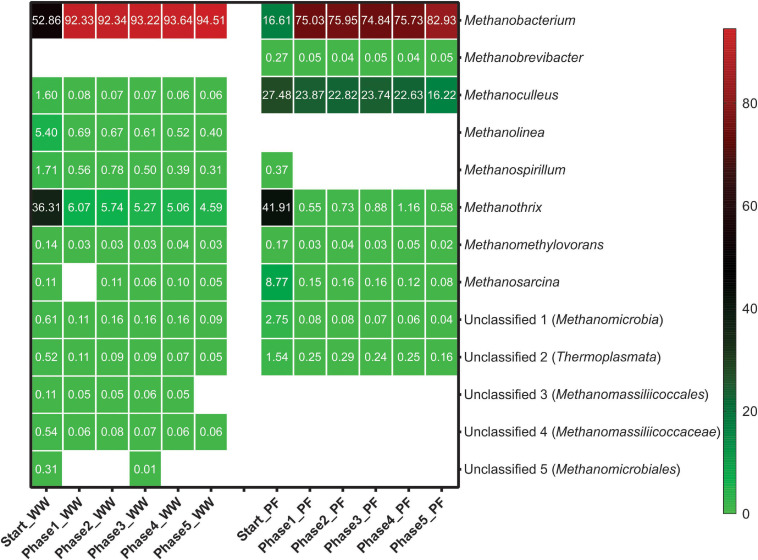
Methanogenic community structure based on *mcr*A gene amplicon sequencing in different phases at genus level. Taxa with relative abundances less than 0.1% were filtered out. Numbers represent the relative abundance in percent and blank space indicates the absence of the respective taxon. Mean values of three biological replicates are presented for Start whereas four biological replicates were averaged for phase 1–5. WW: reactors (inoculated with anaerobic granular sludge from an industrial-scale UASB reactor treating wastewater from paper industry) and PF: reactors (inoculated with digestate from a pilot-scale plug-flow reactor digesting cow manure and corn silage).

The relative abundance of various methanogens throughout the experiment is presented in Figure 5. Hydrogenotrophic methanogenesis was the main pathway in the WW reactors as hydrogenotrophic genera dominated the methanogenic community with ∼62%. The most dominant genera in the WW inoculum were *Methanobacterium* (53%) followed by *Methanothrix* (36%), which suggests that also acetotrophic methanogenesis was active in this inoculum. In contrast, acetotrophic methanogenesis was the main pathway in the PF inoculum as *Methanothrix* (42%) was most dominant together with the versatile genus *Methanosarcina* (9%), which together summed up to more than 50% of all methanogens. In PF, *Methanoculleus* (27%) was the predominant hydrogenotrophic genus together with *Methanobacterium* (17%), whereas *Methanoculleus* (<1%) was underrepresented in WW. H_2_/CO_2_ fed-batch feeding favored *Methanobacterium* in all experimental phases with mean relative abundances of 93 and 77% in WW and PF, respectively. Other hydrogenotrophic methanogens (*Methanolinea*, *Methanospirillum*, and *Methanoculleus*) were strongly outcompeted by *Methanobacterium* in case of WW. The situation was different for PF where two hydrogenotrophic genera (*Methanobacterium* and *Methanoculleus*) dominated from the beginning and stayed predominant throughout the experiment, even though *Methanobacterium* was favored as well over *Methanoculleus.* The versatile genus *Methanosarcina* was outcompeted by strict hydrogenotrophic methanogens in PF likely due to its lower H_2_ uptake rate (Feist et al., 2006; Goyal et al., 2015). However, *Methanosarcina* was still present in both communities until the end of the experiment at very low relative abundance. Our results are consistent with previous studies on H_2_ biomethanation reporting the dominance of *Methanobacteriales* (Kougias et al., 2017; Rachbauer et al., 2017; Savvas et al., 2017). This indicates that methanogens affiliated to the *Methanobacteriales* have a selective advantage over other hydrogenotrophic methanogens in *ex situ* biomethanation. The relative abundance of *Methanothrix* decreased drastically in the end of phase 1 and then remained fairly stable in both communities. The persistence of this obligate acetotrophic throughout the entire experiment is remarkable considering the simple substrate (H_2_/CO_2_) supply and suggests homoacetogenesis as a competing hydrogenotrophic reaction. *Methanothrix* decreased in abundance from 36 to 5% and from 42% to less than 1% in WW and PF, respectively. Although the relative abundance was low, its presence could explain the low acetate concentrations (Table 1). The better performance of WW over PF may be explained by the fact that *Methanobacterium* was dominant initially and throughout the experiment. Hence, selecting an inoculum dominated by *Methanobacteriales* could be advantageous from the application point of view.

Regarding the bacterial community, WW and PF presented distinct community structures that reflected the origin of the inocula (Supplementary Figure 4). The most abundant genera at the start were T78 (*Chloroflexi*) (9 ± 4%) in WW and *Fastidiosipila* (16 ± 5%) in PF. *Petrimonas* was underrepresented in the inocula (<1%) but increased in relative abundance after H_2_/CO_2_ feeding in both communities and remained at comparable levels of around 11% throughout phases 1–5 for WW and PF. *Petrimonas* and *Fastidiosipila*, the latter being dominant throughout all phases in PF, were also found in enrichment cultures fed with H_2_/CO_2_ under similar operating conditions (mode of feeding and mineral medium) (Logroño et al., 2020). So far there are no species described as autotroph for the two aforementioned genera but their enrichment suggests a specific ecological niche in systems fed with H_2_/CO_2_. Further investigations, e.g., isolation or metagenomics, are needed to elucidate their function. When analyzing the top 25 genera, only four genera were shared between WW and PF of which three were unclassified (Supplementary Figure 4). A recent meta-omics study on the genus *Petrimonas* revealed its function in sugar and amino acid fermentation pathways and its widespread occurrence in biogas reactors, particularly in biogas plants with process disorders (Maus et al., 2020). The dominance of this genus in the WW and PF communities might be related to microbial biomass turnover under stress conditions such as substrate shift in phase 1 and starvation in phases 2–5.

At the family level (Supplementary Figure 5), *Anaerolineaceae* (21 ± 3%) in WW and *Ruminococcaceae* (24 ± 4%) in PF dominated at the start. Throughout phases 1–5, *Porphyromonadaceae* remained at comparably high relative abundance in both communities. After H_2_/CO_2_ feeding, families with relative abundances of ≥5% were *Porphyromonadaceae*, *Synergistaceae*, *Anaerolineaceae*, and *Thermotogaceae* in WW, whereas PF was dominated by the families *Porphyromonadaceae*, *Ruminococcaceae*, Unclassified family 5 (*Cloacimonetes*), *Caldicoprobacteraceae*, and MBA03 (*Firmicutes*). The slight production of acetate might be attributed to the presence of *Thermoanaerobacteraceae* (>2%), a family that comprises acetogens (Bengelsdorf et al., 2018). Comparable relative abundances in WW and PF (phase 1–5) were observed. This observation is in accordance with our previous findings (Logroño et al., 2020).

### Effects of Starvation on the Microbial Community Dynamics

**FIGURE 6:**
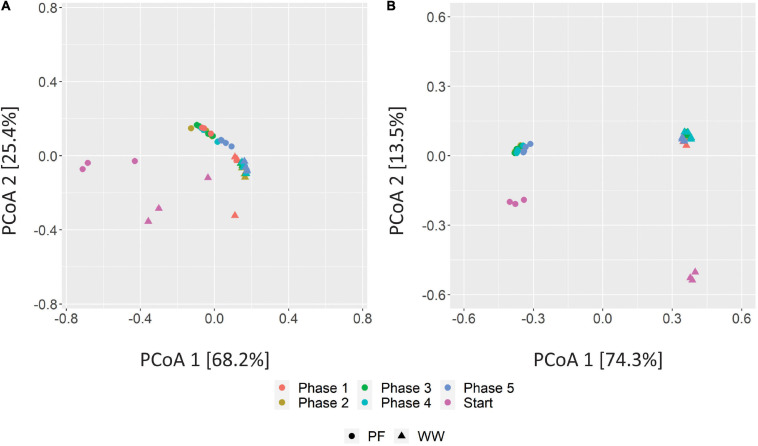
PCoA plots of Bray–Curtis distances of methanogenic **(A)** and bacterial **(B)** communities based on amplicon sequencing of *mcr*A and 16 rRNA genes. Three biological replicates are presented for sludge inoculum (Start) whereas four biological replicates are presented for end of regular fed-batch (phase 1), feeding without shaking (phase 2), and feeding after 7 days (phase 3), 14 days (phase 4) and 21 days (phase 5) of starvation. WW: anaerobic granular sludge from an industrial-scale UASB reactor treating wastewater from paper industry; PF: digestate from a pilot-scale plug-flow reactor digesting cow manure and corn silage.

Principal coordinate analysis of the Bray–Curtis distances between the communities in different phases and from different inocula revealed that the WW samples clustered distinct from PF samples but community specialization toward hydrogenotrophic metabolism was evident after H_2_/CO_2_ feeding (Figure 6). The distinct clustering of the communities can be explained by the different substrates each sludge was originally digesting and likely by the different reactor configuration (UASB vs. plug-flow reactor) as suggested earlier (Mcateer et al., 2020) as well as different operational parameters leading to different process conditions. While the methanogenic communities converged over the phases to one cluster of similar communities for both WW and PF (Figure 6A), the bacterial communities stayed distinct for WW and PF but showed the same trend of community dynamics, reflecting the enrichment of different hydrogenotrophic communities (Figure 6B). In contrast to the strong community shifts caused by H_2_/CO_2_ feeding, the communities did not change much over the phases 1–5. A more detailed PCoA of these samples excluding the start samples is shown in Supplementary Figure 6.

### Implications for P2G Applications in Industrial Wastewater Treatment

**FIGURE 7:**
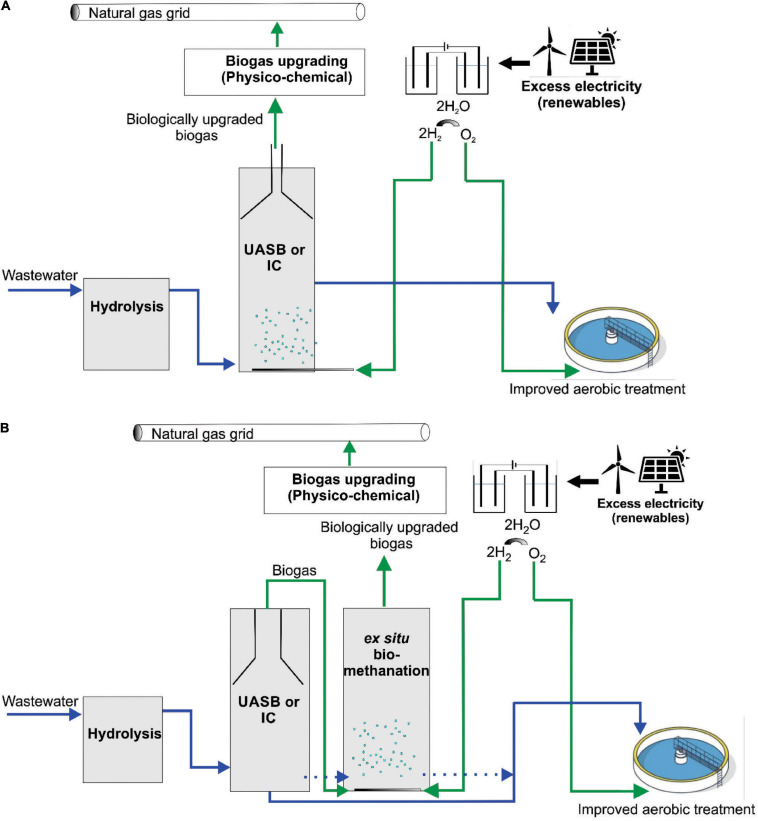
Proposed concept for P2G implementation in wastewater treatment plants of the paper industry. **(A)** *In situ* concept and **(B)** *ex situ* concept. UASB, upflow anaerobic sludge blanket reactor; IC, internal circulation anaerobic reactor.

The results indicated that the WW inoculum led to the enrichment of the best performing community, which was most resilient to starvation disturbances, had the highest hydrogen consumption and methane production rates, and was dominated by hydrogenotrophic methanogens of the genus *Methanobacterium*. Admittedly, considerable differences in terms of hydrogen consumption and methane production rates were only observed in the beginning of phase 1 (Figure 2 and Supplementary Figure 1), which indicates that hydrogenotrophs were more active in WW than in PF from the beginning, in agreement with the dominance of *Methanobacterium* in the WW inoculum. However, the WW community could also better cope with mass transfer limitations (phase 2) and performed slightly better after starvation periods (phases 3–5) as visible in Figure 3. The relative abundance of *Methanobacterium* increased in both communities after repeated starvation periods, which suggests that this genus could endure starvation and ensure efficient biomethanation better than other hydrogenotrophs. On the long run, the PF community might have adapted as reflected by the increasing share of *Methanobacterium*, but the WW inoculum originating from an UASB reactor treating wastewater from paper industry was immediately ready for efficient and resilient biomethanation. Considering the case scenario of a wastewater treatment plant in paper industry that produces biogas and performs biogas upgrading, we propose that biological *ex situ* biomethanation could be easily implemented due to the fact that biocatalytic biomass (granular sludge from an UASB reactor) is readily available from the wastewater treatment process, the reactor infrastructure may be available and oxygen resulting from water electrolysis could be used on site for improving the aerobic treatment step. The approach (Figure 7) could be implemented as *in situ* (partial biogas upgrading through hydrogen injection in the main anaerobic digester), *ex situ* (converting the CO_2_ fraction of the biogas in facilities where physical or chemical biogas upgrading is already in operation) or hybrid approach, where the two aforementioned concepts are combined.

This study shows the effect of starvation on the hydrogen consumption and methane production rates and the microbial community changes in two different inocula that were fed with hydrogen and carbon dioxide. We found that microbial communities are functionally resilient upon starvation disturbances in flexible biomethanation of hydrogen, although long-term effects of repeated or prolonged starvation periods need to be studied in future studies with more replicates and controls. Our results suggest that type and origin of the inoculum, community structure and dominant methanogens are important for process performance. Pre-screening a well performing inoculum is thus essential for functional resilience of flexible biomethanation. It appears that the dominance of the genus *Methanobacterium* was decisive for an efficient *ex situ* biomethanation process since the highest hydrogen consumption rates were observed for the inoculum with this property. We also demonstrated that it is possible to perform H_2_/CO_2_ biomethanation with complex microbiota while avoiding VFA accumulation, which is a relevant aspect for practical implementation. The implementation of the P2G concept in wastewater treatment plants of the paper industry, where biocatalytic biomass is readily available, could be a viable option to reduce the carbon footprint of the paper industry.

## Data Availability Statement

Demultiplexed raw sequence data were submitted to the NCBI Sequence Read Archive (SRA) (http://www.ncbi.nlm.nih.gov/Traces/sra/). The dataset for this study can be found under the study accession number PRJNA623376.

## Author Contributions

WL designed the experiments, analyzed the data, and drafted the manuscript. PK and WL conducted the experiments. DP contributed to the bioinformatic analysis, discussion of the results, and revision of the manuscript. SK and MN contributed to the experimental design, supervised the study, critically discussed the results, and revised the manuscript. HH contributed to the discussion of the results and revision of the manuscript. All authors have read and approved the final manuscript.

## Conflict of Interest

The authors declare that the research was conducted in the absence of any commercial or financial relationships that could be construed as a potential conflict of interest.

## References

[B1] Alitalo A. Niskanen M. Aura E. (2015). Biocatalytic methanation of hydrogen and carbon dioxide in a fixed bed bioreactor. *Bioresour. Technol.* 196 600–605. 10.1016/j.biortech.2015.08.021 26298404

[B2] Angelidaki I. Treu L. Tsapekos P. Luo G. Campanaro S. Wenzel H. (2018). Biogas upgrading and utilization: current status and perspectives. *Biotechnol. Adv.* 36 452–466. 10.1016/j.biotechadv.2018.01.011 29360505

[B3] Bailera M. Lisbona P. Romeo L. M. Espatolero S. (2017). Power to gas projects review: lab, pilot and demo plants for storing renewable energy and CO_2_. *Renew. Sustain. Energy Rev.* 69 292–312. 10.1016/j.rser.2016.11.130

[B4] Bassani I. Kougias P. G. Treu L. Porté H. Campanaro S. Angelidaki I. (2017). Optimization of hydrogen dispersion in thermophilic up-flow reactors for ex situ biogas upgrading. *Bioresour. Technol.* 234 310–319. 10.1016/j.biortech.2017.03.055 28340435

[B5] Bengelsdorf F. R. Beck M. H. Erz C. Hoffmeister S. Karl M. M. Riegler P. (2018). Bacterial anaerobic synthesis gas (syngas) and CO_2_ + H_2_ fermentation. *Adv. Appl. Microbiol.* 103 143–221. 10.1016/bs.aambs.2018.01.002 29914657

[B6] Blanco H. Nijs W. Ruf J. Faaij A. (2018). Potential of power-to-methane in the EU energy transition to a low carbon system using cost optimization. *Appl. Energy* 232 323–340. 10.1016/j.apenergy.2018.08.027

[B7] Bolyen E. Rideout J. R. Dillon M. R. Bokulich N. A. Abnet C. C. Al-Ghalith G. A. (2019). Reproducible, interactive, scalable and extensible microbiome data science using QIIME 2. *Nat. Biotechnol.* 37 852–857. 10.1038/s41587-019-0209-9 31341288 PMC7015180

[B8] Burkhardt M. Busch G. (2013). Methanation of hydrogen and carbon dioxide. *Appl. Energy* 111 74–79. 10.1016/j.apenergy.2013.04.080 25193088

[B9] Burkhardt M. Jordan I. Heinrich S. Behrens J. Ziesche A. Busch G. (2019). Long term and demand-oriented biocatalytic synthesis of highly concentrated methane in a trickle bed reactor. *Appl. Energy* 240 818–826. 10.1016/j.apenergy.2019.02.076

[B10] Burkhardt M. Koschack T. Busch G. (2015). Biocatalytic methanation of hydrogen and carbon dioxide in an anaerobic three-phase system. *Bioresour. Technol.* 178 330–333. 10.1016/j.biortech.2014.08.023 25193088

[B11] Callahan B. J. McMurdie P. J. Rosen M. J. Han A. W. Johnson A. J. A. Holmes S. P. (2016). DADA2: high-resolution sample inference from Illumina amplicon data. *Nat. Methods* 13 581–583. 10.1038/nmeth.3869 27214047 PMC4927377

[B12] Feist A. M. Scholten J. C. M. Palsson B. Brockman F. J. Ideker T. (2006). Modeling methanogenesis with a genome-scale metabolic reconstruction of *Methanosarcina barkeri*. *Mol. Syst. Biol.* 2 1–14. 10.1038/msb4100046 16738551 PMC1681478

[B13] Figeac N. Trably E. Bernet N. Delgenès J.-P. Escudié R. (2020). Temperature and inoculum origin influence the performance of ex-situ biological hydrogen methanation. *Molecules* 25:5665 10.3390/molecules25235665 PMC7730501 33271799

[B14] Fish J. A. Chai B. Wang Q. Sun Y. Brown C. T. Tiedje J. M. (2013). FunGene: the functional gene pipeline and repository. *Front. Microbiol.* 4:291. 10.3389/fmicb.2013.00291 24101916 PMC3787254

[B15] Goyal N. Padhiary M. Karimi I. A. Zhou Z. (2015). Flux measurements and maintenance energy for carbon dioxide utilization by *Methanococcus maripaludis*. *Microb. Cell Fact.* 14 1–9. 10.1186/s12934-015-0336-z 26376868 PMC4573941

[B16] Hattori S. (2008). Syntrophic acetate-oxidizing microbes in methanogenic environments. *Microb. Environ.* 23 118–127. 10.1264/jsme2.23.118 21558697

[B17] Jin Q. Kirk M. F. (2018). pH as a primary control in environmental microbiology: 1. Thermodynamic perspective. *Front. Microbiol.* 6:21 10.3389/fenvs.2018.00021

[B18] Kern T. Theiss J. Röske K. Rother M. (2016). Assessment of hydrogen metabolism in commercial anaerobic digesters. *Appl. Microbiol. Biotechnol.* 100 4699–4710. 10.1007/s00253-016-7436-5 26995607

[B19] Klindworth A. Pruesse E. Schweer T. Peplies J. Quast C. Horn M. (2013). Evaluation of general 16S ribosomal RNA gene PCR primers for classical and next-generation sequencing-based diversity studies. *Nucleic Acids Res.* 41 1–11. 10.1093/nar/gks808 22933715 PMC3592464

[B20] Kougias P. G. Treu L. Benavente D. P. Boe K. Campanaro S. Angelidaki I. (2017). Ex-situ biogas upgrading and enhancement in different reactor systems. *Bioresour. Technol.* 225 429–437. 10.1016/j.biortech.2016.11.124 27931939

[B21] Logroño W. Popp D. Kleinsteuber S. Sträuber H. Harms H. Nikolausz M. (2020). Microbial resource management for ex situ biomethanation of hydrogen at alkaline pH. *Microorganisms* 8:614 10.3390/microorganisms8040614 PMC7232305 32344539

[B22] Lucas R. Harms H. Johst K. Frank K. Kleinsteuber S. (2017). A critical evaluation of ecological indices for the comparative analysis of microbial communities based on molecular datasets. *FEMS Microbiol. Ecol.* 93 1–15. 10.1093/femsec/fiw209 27798064

[B23] Martin M. (2013). Cutadapt removes adapter sequences from high-throughput sequencing reads. *EMBnet. J.* 17 10–12. 10.14806/ej.17.1.200

[B24] Maus I. Tubbesing T. Wibberg D. Heyer R. Hassa J. Tomazetto G. (2020). The role of *Petrimonas mucosa* ING2-E5AT in mesophilic biogas reactor systems as deduced from multiomics analyses. *Microorganisms* 8:2024. 10.3390/microorganisms8122024 33348776 PMC7768429

[B25] Mcateer P. G. Christine A. Thorn C. Mahony T. Abram F. Flaherty V. O. (2020). Reactor configuration influences microbial community structure during high-rate, low-temperature anaerobic treatment of dairy wastewater. *Bioresour. Technol.* 307:123221. 10.1016/j.biortech.2020.123221 32222691

[B26] McIlroy S. J. Kirkegaard R. H. McIlroy B. Nierychlo M. Kristensen J. M. Karst S. M. (2017). MiDAS 2.0: an ecosystem-specific taxonomy and online database for the organisms of wastewater treatment systems expanded for anaerobic digester groups. *Database* 2017:bax016. 10.1093/database/bax016 28365734 PMC5467571

[B27] Oksanen J. Blanchet F. G. Friendly M. Kindt R. Legendre P. McGlinn D. (2019). *Vegan: Community Ecology Package.* Available online at: https://cran.r-project.org/package=vegan (accessed March 30, 2020)

[B28] R Core Team (2019). *R: A Language and Environment for Statistical Computing.* Vienna: R Foundation for Statistical Computing.

[B29] Rachbauer L. Beyer R. Bochmann G. Fuchs W. (2017). Characteristics of adapted hydrogenotrophic community during biomethanation. *Sci. Total Environ.* 595 912–919. 10.1016/j.scitotenv.2017.03.074 28432991

[B30] Rachbauer L. Voitl G. Bochmann G. Fuchs W. (2016). Biological biogas upgrading capacity of a hydrogenotrophic community in a trickle-bed reactor. *Appl. Energy* 180 483–490. 10.1016/j.apenergy.2016.07.109

[B31] Rittmann S. Seifert A. Herwig C. (2015). Essential prerequisites for successful bioprocess development of biological CH_4_ production from CO_2_ and H_2_. *Crit. Rev. Biotechnol.* 35 141–151. 10.3109/07388551.2013.820685 24020504

[B32] Savvas S. Donnelly J. Patterson T. Chong Z. S. Esteves S. R. (2017). Biological methanation of CO_2_ in a novel biofilm plug-flow reactor: a high rate and low parasitic energy process. *Appl. Energy* 202 238–247. 10.1016/j.apenergy.2017.05.134

[B33] Schaaf T. Grünig J. Schuster M. R. Rothenfluh T. Orth A. (2014). Methanation of CO_2_ - storage of renewable energy in a gas distribution system. *Energy Sustain. Soc.* 4 1–14. 10.1186/s13705-014-0029-1

[B34] Schiebahn S. Grube T. Robinius M. Tietze V. Kumar B. Stolten D. (2015). Power to gas: technological overview, systems analysis and economic assessment for a case study in Germany. *Int. J. Hydrogen Energy* 40 4285–4294. 10.1016/j.ijhydene.2015.01.123

[B35] Stams A. J. M. Van Dijk J. B. Dijkema C. Plugge C. M. (1993). Growth of syntrophic propionate-oxidizing bacteria with fumarate in the absence of methanogenic bacteria. *Appl. Environ. Microbiol.* 59, 1114–1119. 16348912 10.1128/aem.59.4.1114-1119.1993 PMC202247

[B36] Steinberg L. M. Regan J. M. (2008). Phylogenetic comparison of the methanogenic communities from an acidic, oligotrophic fen and an anaerobic digester treating municipal wastewater sludge. *Appl. Environ. Microbiol.* 74 6663–6671. 10.1128/AEM.00553-08 18776026 PMC2576706

[B37] Strübing D. Huber B. Lebuhn M. Drewes J. E. Koch K. (2017). High performance biological methanation in a thermophilic anaerobic trickle bed reactor. *Bioresour. Technol.* 245 1176–1183. 10.1016/j.biortech.2017.08.088 28863994

[B38] Strübing D. Moeller A. B. Mößnang B. Lebuhn M. Drewes J. E. Koch K. (2018). Anaerobic thermophilic trickle bed reactor as a promising technology for flexible and demand-oriented H_2_/CO_2_ biomethanation. *Appl. Energy* 232 543–554. 10.1016/j.apenergy.2018.09.225

[B39] Strübing D. Moeller A. B. Mößnang B. Lebuhn M. Drewes J. E. Koch K. (2019). Load change capability of an anaerobic thermophilic trickle bed reactor for dynamic H_2_/CO_2_ biomethanation. *Bioresour. Technol.* 289:121735. 10.1016/j.biortech.2019.121735 31300304

[B40] Ullrich T. Lindner J. Bär K. Mörs F. Graf F. Lemmer A. (2018). Influence of operating pressure on the biological hydrogen methanation in trickle-bed reactors. *Bioresour. Technol.* 247 7–13. 10.1016/j.biortech.2017.09.069 28942208

[B41] Wickham H. (2016). ggplot2: Elegant Graphics for Data Analysis. New York, NY: Springer.

[B42] Yun Y. M. Sung S. Kang S. Kim M. S. Kim D. H. (2017). Enrichment of hydrogenotrophic methanogens by means of gas recycle and its application in biogas upgrading. *Energy* 135 294–302. 10.1016/j.energy.2017.06.133

