# Supplementary material for: Microbial Communities in Flexible Biomethanation of Hydrogen Are Functionally Resilient Upon Starvation
Source: Front Microbiol. 2021 Feb 11;12:619632. doi: 10.3389/fmicb.2021.619632 (PMC7904901; doi:10.3389/fmicb.2021.619632)
Supplement: Supplementary file 1 [file Data_Sheet_1.PDF]

Supplementary Material

Microbial Communities in Flexible Biomethanation of Hydrogen Are Functionally Resilient Upon Starvation

Washington Logroño[1], Denny Popp[1], Marcell Nikolausz[1], Paul Kluge[1], Hauke Harms[1], Sabine Kleinsteuber[1*]

[1]Department of Environmental Microbiology, Helmholtz Centre for Environmental Research – UFZ, 04318 Leipzig, Germany

*** Correspondence:**
Sabine Kleinsteuber
sabine.kleinsteuber@ufz.de

Text S1. Inoculum preparation

A well homogenized sample of 100 g degassed sludge from WW inoculum was manually crushed, mixed with 100 mL mineral medium A (Logroño et al., 2020) and sieved through 400 µm mesh size. The degassed PF inoculum was also sieved through 400 µm mesh size. The procedure lasted less than 30 min and was performed under a nitrogen gas stream. Both inocula had a pH ~8. After sieving, the inocula were placed inside the anaerobic glovebox (atmosphere of 97% N_2 and 3% H_2) to prepare a master inoculum mixture prior to dispensing to each replicate reactor. Each inoculum (45 mL of WW or PF, respectively) was mixed with 405 mL mineral medium A and stirred at 750 rpm for 10 min to obtain a well homogenized master inoculum mixture. While stirring at 250 rpm inside the glovebox, 50 mL of the master inoculum mixtures were sampled and dispensed to the replicate serum bottles. The bottles were closed with black butyl rubber stoppers (LABSOLUTE, Th. Geyer GmbH, Germany) inside the glovebox and sealed with aluminum caps.

Text S2: Media composition

The anoxic and sterile component 1 (**Table S1**) was supplemented with stock solutions (**Table S2**) according to the requirements of medium A. Detailed information of stock solutions is presented in **Table S3**.

Table S1. Composition of the medium component 1

**Compound**	**Final concentration (g L^{-1})**
	Medium A
**NH_4Cl**	0.5
**KH_2PO_4**	0.2
**$MgCl_2 \times 6\ H_2O$**	0.1
**KCl**	0.2
**NaCl**	2.0
**Yeast extract**	0.2
**Resazurin**	0.0005
**Trace elements SL10 (mL L^{-1})**	1

Note:
Component 1 (850 mL) was made anoxic by stirring in the anaerobic chamber (97% N_2, 3% H_2) for 45 min. Sterilization was done by autoclaving at 121°C for 20 min.

Table S2. Stock solutions used to supplement the media for different experiments

Stock solutions	Volume added (mL L^{-1})
	Medium A
**Selenite-tungstate solution DSM 385 (1:4 diluted)**	4
**Na_2CO_3 (29.41 g L^{-1})**	34
**$NaHCO_3$ (76.00 g L^{-1})**	100
**Cysteine-HCl (30.00 g L^{-1})**	12

Note: Every stock solution was made anoxic by stirring in the anaerobic chamber for 30 min sterilized by filtration (pore size 0.2 µm, Ø 25 mm; LABSOLUTE, Th. Geyer GmbH, Germany).

Table S3. Composition of stock solutions

Component	Concentration (mg L^{-1})
**Trace elements SL10 (DSMZ medium 320)**	
**$FeCl_2 \times 4\ H_2O$**	1500
**$ZnCl_2$**	70
**$MnCl_2 \times 4\ H_2O$**	100
**H_3BO_3**	6
**$CoCl_2 \times 6\ H_2O$**	190
**$CuCl_2 \times 2\ H_2O$**	2
**$NiCl_2 \times 6\ H_2O$**	24
**$Na_2MoO_4 \times 2\ H_2O$**	36
**Selenite-tungstate solution (DSMZ medium 385)**	
**NaOH**	500
**Na_2SeO_3 x 5 H_2O**	3.0
**Na_2WO_4 x 2 H_2O**	4.0

Note: For preparing SL10, $FeCl_2$ was first dissolved in HCl (25%, 10 mL L^{-1}) and then diluted in water. Subsequently, other salts were added and dissolved. The 1:4 dilution was prepared with anoxic water and filter-sterilized after 30 min stirring in the anaerobic chamber.

References

Logroño, W., Popp, D., Kleinsteuber, S., Sträuber, H., Harms, H., and Nikolausz, M. (2020). Microbial resource management for ex situ biomethanation of hydrogen at alkaline pH. *Microorganisms 8*, 614. doi: 10.3390/microorganisms8040614.

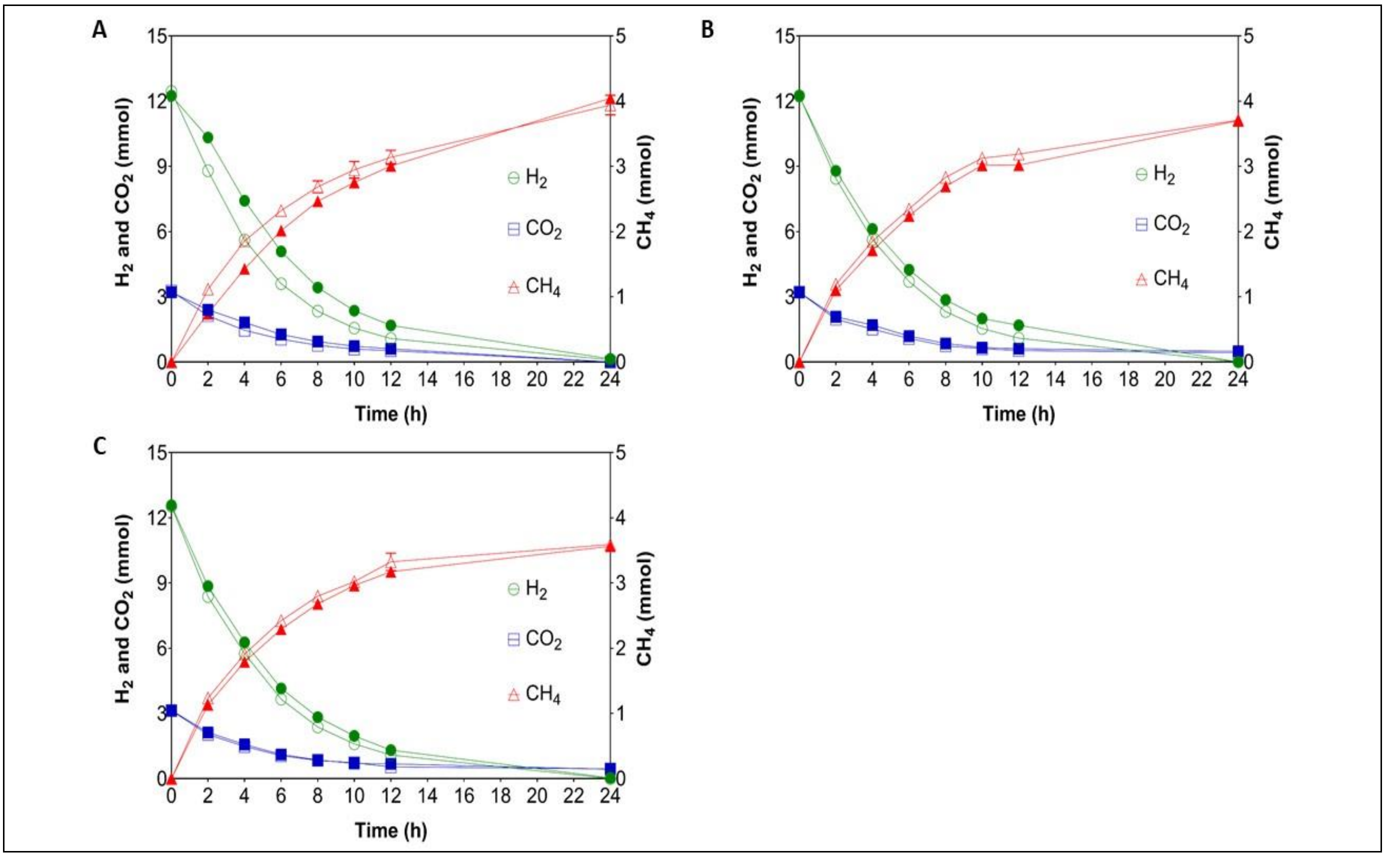

Figure S1. Gas consumption (H_2 and CO_2) and production (CH_4) rates in phase 1 (regular fed-batch feeding) measured on three sampling days (A: day 7, B: day 21 and C: day 53). Open symbols: reactors WW (inoculated with anaerobic granular sludge from an industrial-scale UASB reactor treating wastewater from paper industry); filled symbols: reactors PF (inoculated with digestate from a pilot-scale plug flow reactor digesting cow manure and corn silage). The mean and standard deviation of n=4 are depicted.

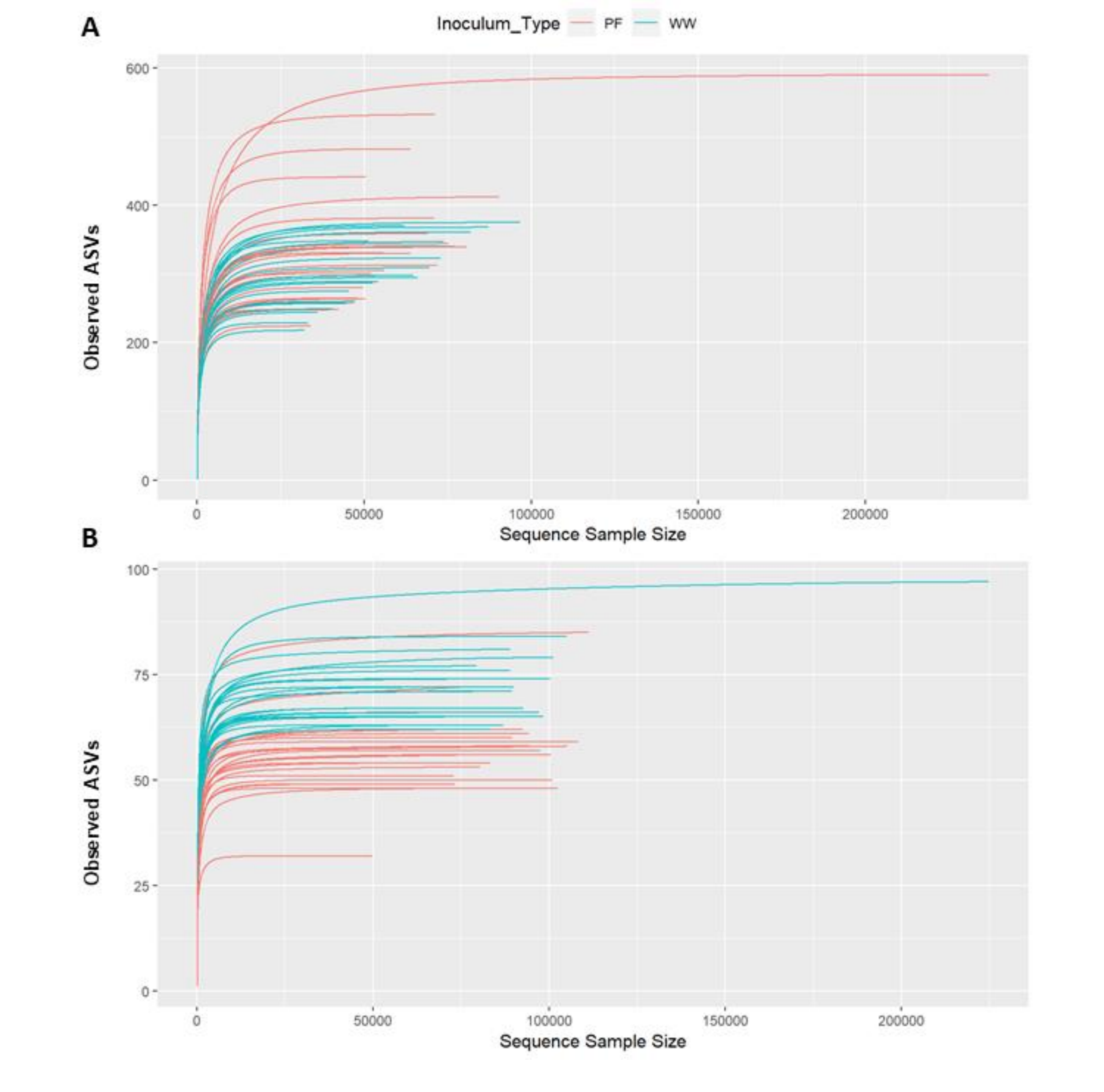

Figure S2. Rarefaction curves showing the observed ASVs of the bacterial communities (A) and the methanogenic communities (B) of WW reactors (inoculated with anaerobic granular sludge from an industrial-scale UASB reactor treating wastewater from paper industry) and PF reactors (inoculated with digestate from a pilot-scale plug flow reactor digesting cow manure and corn silage). Each curve represents the independent sequenced samples and color refers to each inoculum.

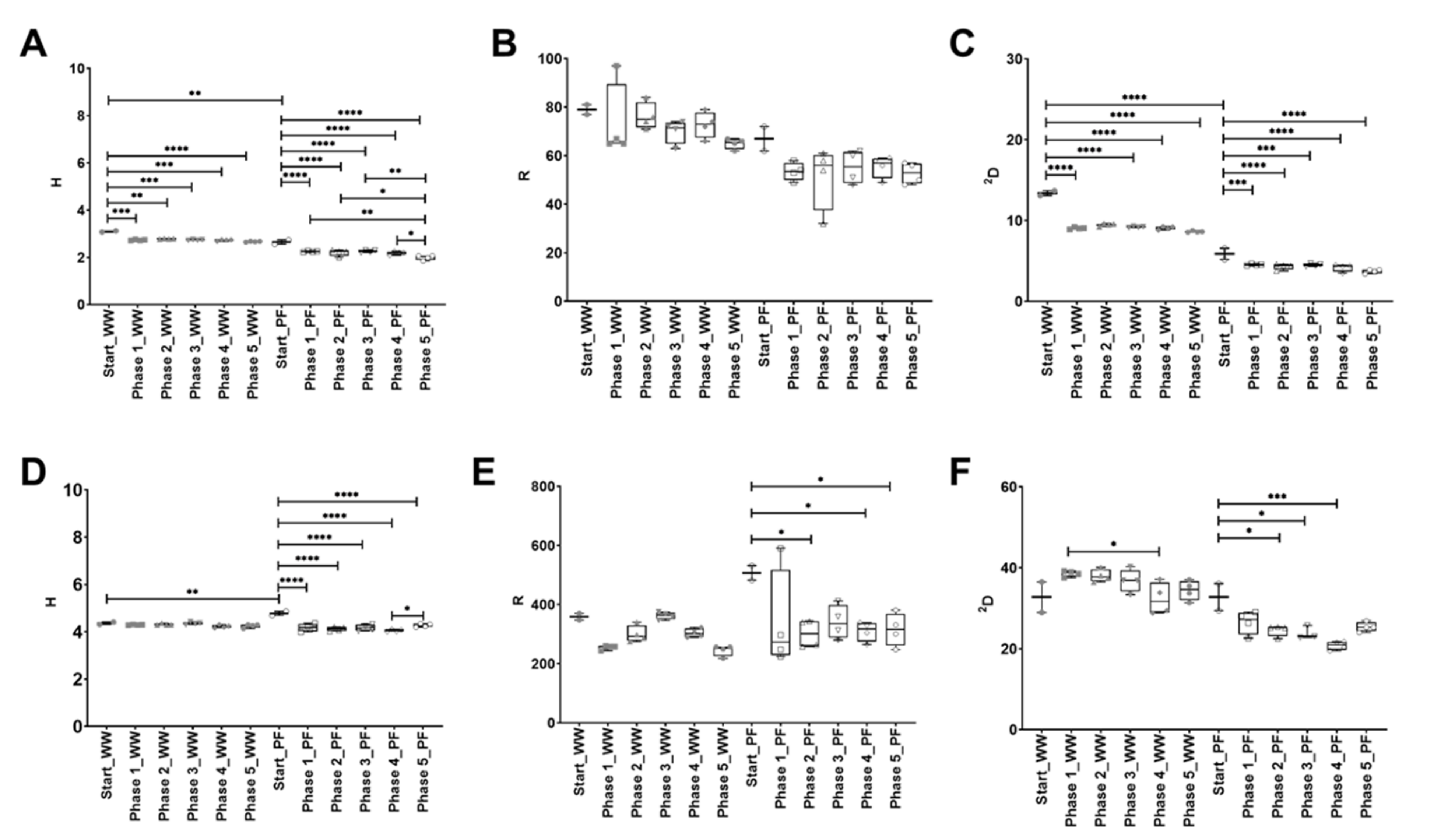
A
B
C
D
E
F
H
R
²D
Start_WW
Phase 1_WW
Phase 2_WW
Phase 3_WW
Phase 4_WW
Phase 5_WW
Start_PF
Phase 1_PF
Phase 2_PF
Phase 3_PF
Phase 4_PF
Phase 5_PF

Figure S3. Box plots of Shannon index (H), richness (R) and diversity for q=2 (^{2}D) in different phases. Methanogenic communities (A, B and C) bacterial communities (D, E and F). Significant differences between groups are indicated with * when $p < 0.05$. Note that for start n=2 whereas for phases 1 to 5 n=4. WW: reactors inoculated with anaerobic granular sludge from an industrial-scale UASB reactor treating wastewater from paper industry; PF: reactors inoculated with digestate from a pilot-scale plug flow reactor digesting cow manure and corn silage.

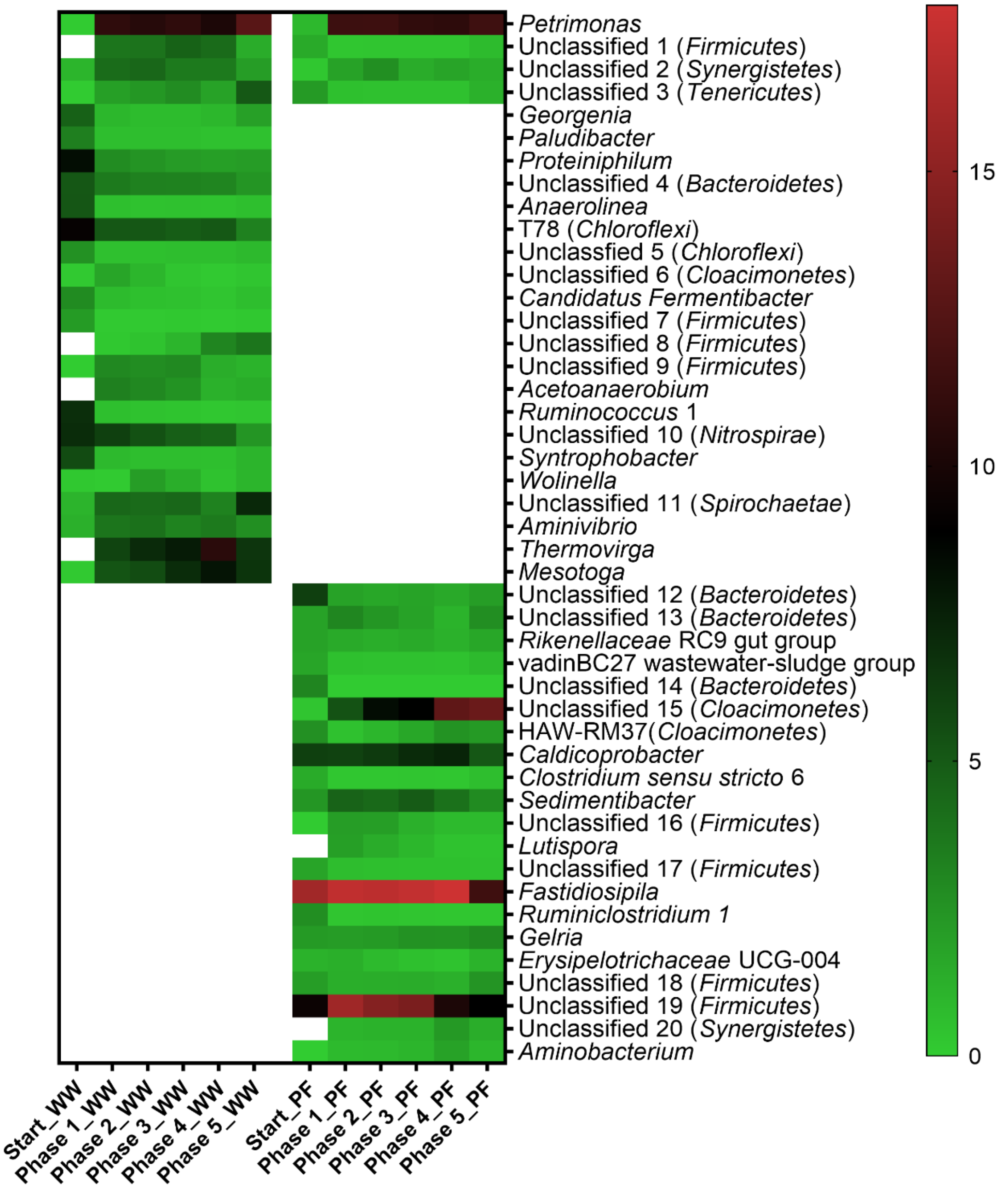

Figure S4. Bacterial community structure in different phases (top 25 bacterial genera). Taxa with relative abundances less than 0.01% were filtered out. Blank space indicates the absence of the respective taxon. Mean values of three biological replicates are presented for Start whereas four biological replicates were averaged for phase 1-5. WW (anaerobic granular sludge from an industrial-scale UASB reactor treating wastewater from paper industry) and PF (digestate from a pilot-scale plug flow reactor digesting cow manure and corn silage).

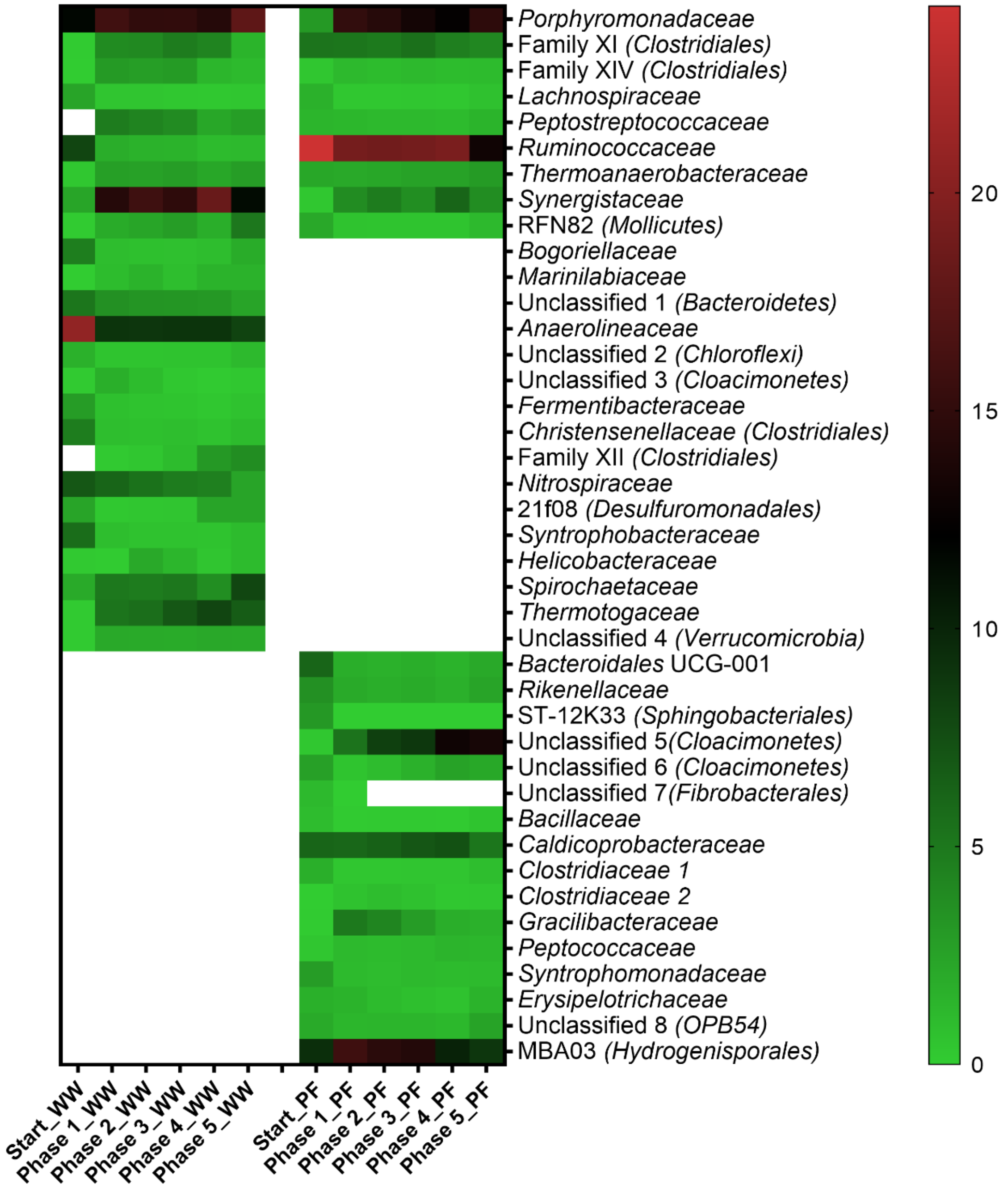

Figure S5. Bacterial community structure in different phases (top 25 bacterial families). Taxa with relative abundances less than 0.01% were filtered out. Blank space indicates the absence of the respective taxon. Mean values of three biological replicates are presented for Start whereas four biological replicates were averaged for phase 1-5. WW (anaerobic granular sludge from an industrial-scale UASB reactor treating wastewater from paper industry) and PF (digestate from a pilot-scale plug flow reactor digesting cow manure and corn silage).

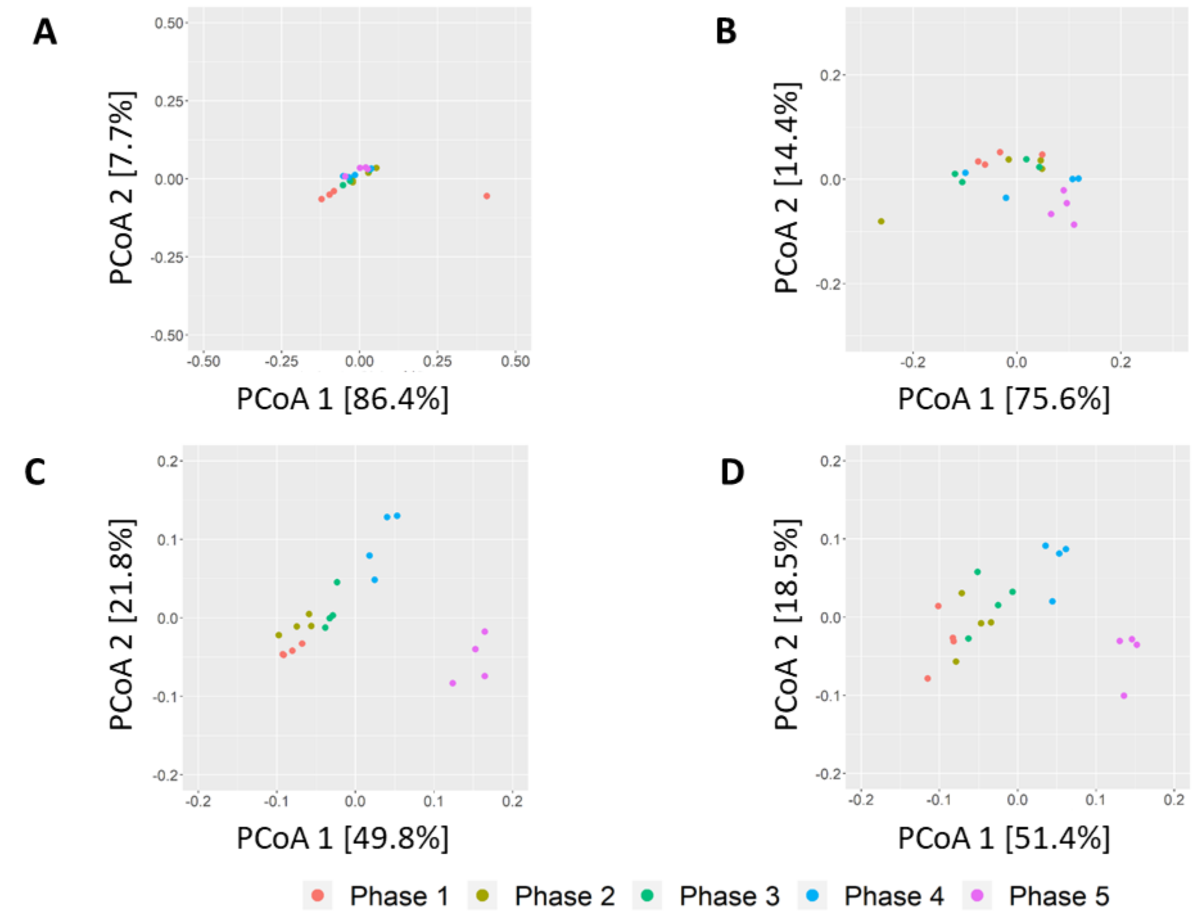

Figure S6. Principal coordinate analysis (PCoA) based on Bray–Curtis distances of microbial community structures excluding the start inoculum. Four biological replicates are presented for phase 1-5. Methanogenic communities is depicted in A (WW) and B (PF) and bacterial communities is depicted in C (WW) and D (PF). WW (anaerobic granular sludge from an industrial-scale UASB reactor treating wastewater from paper industry) and PF (digestate from a pilot-scale plug flow reactor digesting cow manure and corn silage).

We plotted the Bray-Curtis distances between samples without considering the inoculum to explore in more detail how the different starvation events influenced the community structure. Further, we applied the analysis of similarities (ANOSIM) on the dissimilarity matrix to test if the differences in community structures could be explained by the experimental phases (phases 1 to 5). The methanogenic community differences could be explained by the experimental phases for both inocula (WW: $p = 0.0024$ and PF: $p = 0.0141$) (**Figure S7 A and B**). Likewise for the bacterial communities (WW: $p = 1e\text{-}04$ and PF: $p = 1e\text{-}04$) (**Figure S7 C and D**). This suggests that the starvation events changed the microbial community structures when compared to the regular fed-batch feeding, although the time effect on the community structure cannot be ruled out.
